# Effects of Soil Restoration in Cadmium-Polluted Areas on Body Cadmium Burden and Renal Tubular Damage in Inhabitants in Japan

**DOI:** 10.3390/toxics13121010

**Published:** 2025-11-22

**Authors:** Kazuhiro Nogawa, Masaru Sakurai, Yuuka Watanabe, Masao Ishizaki, Yasumitsu Ogra, Yu-Ki Tanaka, Hirotaro Iwase, Kayo Tanaka, Teruhiko Kido, Hideaki Nakagawa, Yasushi Suwazono, Koji Nogawa

**Affiliations:** 1 Department of Occupational and Environmental Medicine, Graduate School of Medicine, Chiba University, Chiba 260-8670, Japan; nogawa@chiba-u.jp (K.N.); watanabe155@chiba-u.jp (Y.W.);; 2 Department of Social and Environmental Medicine, Kanazawa Medical University, Kanazawa 920-0293, Japan; m-sakura@kanazawa-med.ac.jp (M.S.); issa1@kanazawa-med.ac.jp (M.I.); hnakagaw@kanazawa-med.ac.jp (H.N.); 3 Graduate School of Pharmaceutical Sciences, Chiba University, Chiba 260-8670, Japan; ogra@chiba-u.jp (Y.O.); yu-ki.tanaka@chiba-u.jp (Y.-K.T.); 4 Department of Legal Medicine, Graduate School of Medicine, Chiba University, Chiba 260-8670, Japan; iwase@faculty.chiba-u.jp (H.I.); kayo-t4711@chiba-u.jp (K.T.); 5 Faculty of Health Sciences, Institute of Medical Pharmaceutical and Health Sciences, Kanazawa University, Kanazawa 920-1192, Japan; tkido@staff.kanazawa-u.ac.jp

**Keywords:** lifetime cadmium intake, urinary cadmium, β2-microglobulin, soil restoration

## Abstract

This study explored the effects of soil restoration on cadmium (Cd) body burden and renal tubular damage in inhabitants of Cd-polluted areas by estimating the lifetime Cd (LCd) intake and expected LCd intake without soil restoration. In total, 1819 participants (991 men and 828 women) were included in the analysis. Furthermore, 845 participants (503 men and 342 women) who had lived in Cd-polluted areas before soil restoration were selected to estimate LCd intake with and without soil restoration. LCd intake was estimated based on residential history and rice Cd concentrations in each area. First morning urine samples were collected for urinary Cd (U-Cd, as the Cd body burden) and β2-microglobulin (as the renal tubular marker) measurements. The mean LCd intake was 3.0 g for men and 2.6 g for women in Cd-polluted areas with soil restoration. The mean expected LCd intake without soil restoration was 5.1 g for men and 4.6 g for women, indicating that soil restoration reduced LCd intake by approximately 2 g for both sexes. Soil restoration significantly reduces LCd intake, Cd body burden, and renal tubular effects. This information is crucial for developing strategies to reduce Cd exposure worldwide.

## 1. Introduction

Itai-Itai disease was first recognized in the Jinzu River basin around 1920 [1]. The affected patients were typically middle-aged or older women who experienced severe pain and suffered bone fractures with minimal trauma [1,2]. Cadmium (Cd) released from a mine upstream had contaminated the soil in the region’s paddy fields. Between 1971 and 1976, the Toyama prefectural authorities conducted a comprehensive assessment of Cd concentrations in soil and rice grains within the Jinzu River basin [3,4]. A survey of 1600 soil samples revealed Cd levels ranging from 0.21 to 9.54 ppm, resulting in an average concentration of 1.37 ppm. This was four times higher than the mean of 0.35 ppm observed in 77 soil samples taken from unpolluted areas [3,4]. Analysis of unpolished rice samples revealed a broad spectrum of Cd concentrations ranging from 0.00 to 5.20 ppm. The resulting mean concentration of Cd was 0.37 ppm, which is 2.5 times higher than the average of 0.15 ppm measured in 77 samples collected from fields deemed non-polluted [3,4]. Thus, Cd concentrations in the rice harvested from these fields were significantly elevated, leading to high levels of environmental Cd exposure in residents who consumed the rice. This excessive Cd exposure disrupted bone mineral metabolism, resulting in Itai-Itai disease, characterized by kidney dysfunction, osteomalacia, and osteoporosis [1,2,3,5]. In response, strategies were implemented to reduce Cd exposure in the Jinzu River basin. At that time, residents used water from the Jinzu River for cooking and other daily activities. To mitigate Cd exposure from water, waterworks were constructed by 1974. Additionally, in 1970, the Japanese government set a regulatory limit for Cd concentrations in brown rice to prevent further Cd exposure through dietary sources. To restore rice production in the Jinzu River basin, Toyama Prefecture identified over 1600 hectares of paddy fields that required Cd decontamination. Soil restoration efforts began in the upstream areas of the Jinzu River and were completed by March 2012. Depending on the condition of the paddy field, the methods of “soil dressing after burying the polluted top soil” or “soil dressing directly onto the polluted top soil” were employed [3,6]. Today, Cd levels in brown rice from restored areas are well below the regulatory limits, and the region’s farmlands have been rehabilitated, providing both rich and safe agricultural land [3].

We first evaluated the changes in Cd body burden and renal tubular markers in residents of the Cd-polluted area following soil restoration efforts [7]. Among the participants, urinary Cd (U-Cd) concentrations significantly decreased in both men and women after the restoration. A similar trend was observed in the urinary β2-microglobulin (U-β2MG) concentrations in both genders. To better understand this reduction, it is essential to evaluate each individual’s Cd intake prior to soil restoration and after restoration, as well as to assess how total lifetime Cd (LCd) intake has changed in these areas [7]. Meanwhile, Cd pollution remains a significant issue in countries such as China and Thailand, where Cd exposure continues to pose health risks to the general population [8,9,10,11]. Consequently, information on the effectiveness of soil restoration in mitigating Cd contamination in polluted areas is crucial for addressing this ongoing problem.

The aim of this study was to clarify the impact of soil restoration on Cd body burden and renal tubular damage in residents of Cd-polluted areas. This was achieved by estimating LCd intake, predicting LCd intake without soil restoration, and analyzing these values according to current renal tubular effects.

## 2. Materials and Methods

### 2.1. Participants

The participants in this study were residents from Cd-polluted areas in the Jinzu River basin, Toyama Prefecture, Japan. Briefing sessions about the survey were conducted for the general population across six districts, including areas where soil restoration had taken place. Households in the target areas were invited to participate in the survey through written invitations. In terms of participant recruitment, we initially invited all residents of the six target areas affected by past cadmium contamination to attend an information meeting about the survey. Of those, 2118 (1105 men and 1013 women) were invited to participate in the study, of whom 2012 (95.0%; 1051 men and 961 women) took part in the health surveys conducted in 2020. In the analysis of the relationship between years of residence and urinary variables, 193 participants (60 men and 133 women) with incomplete residential history data were excluded. Therefore, 1819 participants (991 men and 828 women) were included in the analysis. Furthermore, 845 participants (503 men and 342 women) who had lived in the polluted areas prior to soil restoration were selected in the analysis to estimate Cd exposure in the absence of soil restoration.

**Figure 1:**
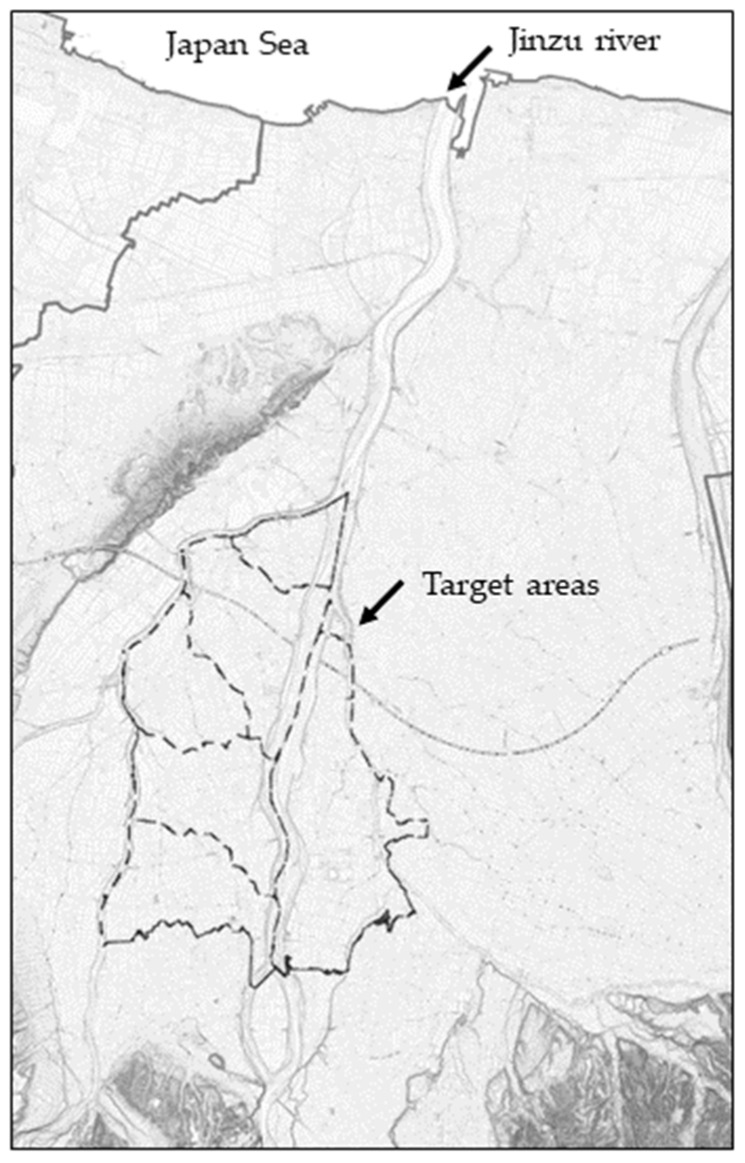
The map of study areas. Source: Historical Administrative Boundary Dataset (Beta Version, Created by CODH, doi:10.20676/00000447), modified by the authors.

Written informed consent was obtained from all participants. This study was approved by the Ethics Committee of Kanazawa University (no. 968-I) and the Ethics Committee of Kanazawa Medical University (nos. I 484 and I 750). The map of the study areas is shown in Figure 1.

### 2.2. Data Collection

For sample collection, urine containers and questionnaires were mailed to the target participants. The first morning urine sample was collected in spring and at the end of 2020 by the participants and submitted to the community center. In the laboratory, urine specimens were stored frozen. Following oxidative digestion, U-Cd concentrations were easured using inductively coupled plasma mass spectrometry (ICP-MS; 8800 Triple Quadrupole ICP-MS; Agilent Technologies, Santa Clara, CA, USA). As a preliminary experiment, the production rate of molybdenum oxide was investigated in relation to the concentration of molybdenum using the molybdenum standard solutions, and a regression equation was obtained. During the measurement of U-Cd, the concentration of molybdenum was determined simultaneously and the degree of mass spectrometric interference by molybdenum oxide was estimated using the regression equation to correct for interference in U-Cd concentration. The detection limit of U-Cd was 0.04 μg/L. Cd was detected in all urine samples. U-β2MG levels were determined by latex agglutination, and creatinine (Cr) levels were measured using an enzymatic method (BML Inc., Tokyo, Japan). The questionnaire collected data on the participants’ gender, age, full residential history (from birth to 2020), and smoking status (non-smoker, ex-smoker, or smoker).

### 2.3. Lifetime Cd Intake

Between 1971 and 1976, the Toyama Prefectural Department of Health and Welfare [12] measured Cd concentrations in unpolished non-glutinous rice across the entire endemic region of the Jinzu River basin and in adjacent water systems. In total, 2446 rice samples were analyzed from 88 hamlets. Details of the rice collection and measurement methods are described in previous publications [12,13]. Using full residential history data, Cd intake for each period of residence was calculated based on Nogawa’s formula [14]: (mean Cd concentration in rice of the present hamlet × 333.5 g/day + 34 μg/day) × 365 days/year × number of years of residence in the present hamlet + 50 μg/day × 365 days/year × number of years living in Cd non-polluted regions. In this formula, 333.5 g/day represents the average daily rice intake in the region in 1970, 34 µg/day is the Cd intake from foods other than rice, and 50 µg/day is the average Cd intake in non-polluted areas of Japan. All places of residence and length of residence since birth are interviewed. Combining that information with the time of soil restoration at each area, years of residence in each Cd-contaminated area was determined. For each period of residence, the Cd concentration in rice from the corresponding contaminated area was used in the formula. Furthermore, Cd intake from each residence period up to 2020 was summed to calculate LCd intake. The formula was also applied to estimate Cd intake assuming no soil remediation had taken place. To assess the impact of soil restoration, expected LCd intake without soil restoration was estimated, assuming that the rice Cd concentrations remained constant until 2020, and this was compared with the current LCd intake.

### 2.4. Statistical Analysis

Cr adjustment was applied to the U-Cd and U-β2MG data. These variables were then log-transformed for analysis. First, multiple regression analysis was conducted for all participants, with LCd intake and smoking status as independent variables, and the log-transformed U-Cd and U-β2MG as the dependent variables. Variable selection was performed using the stepwise method.

Next, to assess the effect of soil restoration, the obtained regression model for LCd intake was used to estimate the expected log-transformed U-Cd and U-β2MG levels, assuming no soil restoration. These estimates were based on the calculated expected LCd intake without soil restoration, as well as smoking status, focusing on participants with a history of living in contaminated hamlets. The expected values of LCd intake, U-Cd, and U-β2MG (without soil restoration) and the current measured values were then compared using paired *t*-tests.

To further confirm the association between renal tubular damage and LCd intake, logistic regression analysis was performed. Renal tubular damage was classified as either initial damage (U-β2MG ≥ 300 µg/g Cr) or clinical damage (U-β2MG ≥ 1000 µg/g Cr) and used as the dependent variable. LCd intake and smoking status were included as independent variables. Model selection was conducted using the forward elimination method. Statistical analyses were performed using SPSS software (version 22; IBM Corp., Armonk, NY, USA), with *p*-values < 0.05 considered statistically significant.

## 3. Results

The characteristics of participants included in the regression model of U-Cd and U-β2MG are presented in Table 1. The arithmetic mean LCd intake values were 2.07 g in men and 1.71 g in women. The geometric mean of U-Cd was 0.98 μg/g Cr in men and 1.53 μg/g Cr in women. The geometric mean of U-β2MG was 174 μg/g Cr in men and 200 μg/g Cr in women.

Table 2 presents the regression coefficients for an increase of 1 g in LCd intake in relation to log-transformed U-Cd and U-β2MG. LCd intake showed a consistently significantly association with increases in U-Cd and U-β2MG in men and women, respectively.

Table 3 presents the expected LCd intake, U-Cd, and U-β2MG, assuming no soil restoration, and comparison with the actual levels. The mean expected LCd intake was 5.06 g in men and 4.57 g in women. The current LCd intake in these participants was estimated to be 2.99 g in men and 2.59 g in women, resulting in a significant reduction in LCd intake of approximately 2 g. The mean expected U-Cd without soil restoration was 2.18 µg/g Cr in men and 4.22 µg/g Cr in women. In contrast, the measured U-Cd after soil restoration was 1.23 µg/g Cr in men and 1.99 µg/g Cr in women, indicating a significant decrease of 50–60%. The mean expected U-β2MG without soil restoration was 430 µg/g Cr in men and 646 µg/g Cr in women. After soil restoration, the measured U-β2MG levels were 213 µg/g Cr in men and 256 µg/g Cr in women, reflecting a significant reduction of 40–50%.

Table 4 presents the odds ratios and 95% confidence intervals for LCd intake associated with β2-microglobulinuria in all participants. The odds ratios for an increase of 1 g in LCd intake for U-β2MG levels ≥ 300 µg/g Cr were 1.75 in men and 1.84 in women. For U-β2MG levels ≥ 1000 µg/g Cr, the odds ratios were 1.71 in men and 2.15 in women. These results indicate that increased LCd intake is significantly associated with a higher risk of both initial (U-β2MG ≥ 300 µg/g Cr) and clinical (U-β2MG ≥ 1000 µg/g Cr) renal tubular damage in both men and women.

## 4. Discussion

To summarize the main results of the study, the LCd intake reflecting the completion of soil restoration was 2.99 g for men and 2.59 g for women among participants who had lived in Cd-polluted areas with soil restoration. In contrast, the expected LCd intake assuming without soil restoration was 5.06 g for men and 4.57 g for women, indicating that soil restoration has reduced Cd intake by approximately 2 g for both sexes.

It was estimated that U-Cd, as an indicator of current Cd body burden, could be reduced by soil restoration by approximately 50–60% relative to the expected U-Cd levels without restoration. Similarly, U-β2MG, serving as an indicator of tubular effects, was significantly suppressed by soil restoration by 40–50% relative to the expected U-β2MG levels without restoration. These findings suggest that soil restoration has contributed to a substantial reduction in Cd intake, body burden, and the health effects associated with Cd exposure among the inhabitants of these polluted areas. To our knowledge, no previous study has conducted such an estimation. Furthermore, in this study, U-β2MG was utilized as an indicator of renal tubular damage. Previous research has reported that higher U-β2MG concentrations are associated with increased mortality rates [15,16,17,18,19]. Therefore, the reduction in renal tubular damage indicates a potential decrease in mortality among residents in these Cd-polluted areas.

In addition to the kidney damage examined in this study, there are other known long-term health effects of Cd exposure [2]. Occupational exposure to Cd primarily affects the respiratory system through inhalation. Acute exposure can cause pulmonary edema and pneumonia, while chronic exposure is known to cause chronic obstructive pulmonary disease [2]. Furthermore, while severe cases are known to cause osteomalacia and osteoporosis, as seen in Itai-Itai disease, even exposure levels from the general environment have been reported to cause reduced bone density and increased fracture risk [20,21]. Regarding carcinogenicity, associations have been reported between occupational exposure and lung cancer in workers, and among the general population, prostate cancer [22] in men and ovarian cancer [23], breast cancer [24], and endometrial cancer [25] in women. The International Agency for Research on Cancer (IARC) has classified Cd as a human carcinogen, categorizing it within Group 1 of the IARC classification system [26]. However, some studies could not identify the health effect of cadmium intake [27,28,29].

In this study, we adopted LCd intake as an indicator of Cd exposure to clarify the differences in body burden with and without soil restoration. It has been reported that LCd intake is strongly correlated with U-Cd, which serves as an indicator of internal exposure to Cd [30,31]. These reports should be mentioned to demonstrate the accuracy of LCd intake. Our research group has conducted studies using LCd intake and has identified significant relationships between LCd intake and health effect indicators [32,33]. In China, a report using the benchmark dose approach indicated reference levels for food-derived Cd intake concerning renal damage [9]. The benchmark dose low for food-derived Cd intake was reported as 1–2 g, close to the benchmark dose low in the only previous report [33]. In the present study, we also found a strong correlation between LCd intake and U-Cd, further validating LCd intake as an accurate indicator of Cd exposure. Then, as shown in Table 3, U-Cd and U-β2MG levels without soil restoration were estimated for each participant using the obtained regression equations.

Long-term changes in U-Cd following measures to reduce Cd exposure from rice consumption have been reported in both China and Japan. In China, an 8-year follow-up study [34] was conducted among residents (*n* = 142) in three areas of Zhejiang Province: Jiaoweibao (highly polluted), Nanbaixiang (moderately polluted), and Yantou (nonpolluted). In 1995, the Cd concentration of rice produced in the highly polluted area reached as high as 3.7 mg Cd/kg, prompting local health authorities to recommend that residents consume rice from nonpolluted areas starting in 1996. By 1998, the U-Cd levels were 1.79 µg/g Cr in the nonpolluted area, 3.62 µg/g Cr in the moderately polluted area, and 11.6 µg/g Cr in the highly polluted area. By 2006, U-Cd levels increased to 2.31 µg/g Cr in the nonpolluted area and 3.79 µg/g Cr in the moderately polluted area, while the highly polluted area saw a decrease to 8.97 µg/g Cr. Thus, a reduction in U-Cd was confirmed only in the highly polluted area. Regarding renal effect markers, while the increase in urinary albumin showed a degree of reversal, recovery for N-acetyl-β-D-glucosaminidase was incomplete, and there was little evidence of recovery for β2-microglobulin after cessation of exposure to contaminated rice. Therefore, it was demonstrated that identifying improvement in renal tubular damage can be challenging. Many tropical soils in South America, especially andesite soils of volcanic origin, contain naturally high concentrations of Cd [35]. Cultivated crops such as bananas, cocoyams, and cacao accumulate high levels of Cd when grown in Cd-contaminated soils. In the Huánuco region of Peru, the mean Cd concentration in cocoa was reported to be 2.46 mg/kg, ranging from 0.2 to 12.56 mg/kg [35]. Considering the dangers of excess Cd consumption to humans, the Joint FAO/WHO Expert Committee on Food Additives (JECFA) has defined maximum limits (ML) of Cd in cocoa products as 0.3–2.0 mg/kg [35,36]. Furthermore, a report on the distribution of Cd in various European soils revealed the highest concentrations in Ireland, England, the western Alps, southern France, the Belgian-German border, southern Sardinia, eastern Italy, Slovenia, Croatia, Albania, and Greece. Most Cd-enriched areas reflect the legacy of the intensive use of phosphate fertilizers and sewage sludge on land, except in Ireland, southern Sardinia, Poland, and the Goslar district in Germany, where Cd levels are thought to mainly reflect historical Zn–Pb mining [37]. Additionally in Cd-polluted areas of Japan, the cadmium intake levels of female farmers were found to be close to the tolerable weekly intake level [38]. Consequently, exposure to Cd from the general environment remains a significant international concern.

In Japan, a long-term follow-up study on U-Cd concentrations after soil restoration has been reported. Following soil restoration conducted in the Kakehashi River basin in 1981, a subset of residents (*n* = 20 or =28) were monitored to assess their health status. It was found that U-Cd concentrations did not exhibit a marked decrease even 28–30 years post-restoration [39,40]. To our knowledge, there are no other reports on the Cd body burden after soil restoration in Cd-polluted areas. The persistently high U-Cd concentrations in the elderly are likely attributable to significant accumulation of Cd in the body resulting from long-term exposure.

As mentioned earlier, only a few studies have examined changes in Cd burden and health effects resulting from reduced Cd exposure due to soil restoration or the cessation of rice harvesting in Cd-polluted areas. However, a notable strength of this study is that LCd intake was calculated using data on rice Cd concentrations specific to each inhabited area, along with detailed residential history information. This approach allowed for an accurate assessment of changes in Cd intake due to relocation. Thus, this study provides a more precise evaluation of Cd exposure prior to soil restoration. The present study, in conjunction with previous research [7], underscores that reducing Cd exposure through soil restoration is an effective and valuable measure for mitigating the health effects of Cd in residents of Cd-polluted areas.

The target areas of this study are among the most historically significant regions in the world affected by severe Cd exposure, notably resulting in Itai-Itai disease. This region has traditionally been a major rice-growing area, and the local community has actively worked to restore the fields and river waters to their former abundance. Since the identification of Itai-Itai disease, the Toyama prefectural authorities, in collaboration with the community, has been continuously improving the soil in these Cd-polluted areas for over 50 years.

In the present study, we were able to assess LCd intake in greater detail, utilizing urine samples and comprehensive relocation information provided by the inhabitants. This allowed us to evaluate the Cd body burden and markers of renal effects in urine. The biological half-life of Cd is estimated to be 10–30 years [2], and, similar to the carcinogenic risk posed by chemicals, long-term fieldwork is essential for confirming reductions in adverse health effects. The results of this study serve as an important example of effective measures to address long-term health risks and are considered to hold significant public health value. Previously, the Scientific Panel on Contaminants in the Food Chain (CONTAM) of the European Food Safety Authority (EFSA) conducted a meta-analysis of data from 35 studies involving 30,000 individuals [41]. Based on the derived reference point of 4 µg/g Cr of U-Cd, the EFSA CONTAM Panel established a tolerable weekly intake (TWI) of 2.5 µg/kg body weight [41]. Furthermore, the Joint FAO/WHO Expert Committee on Food Additives (JECFA) has also continued to evaluate Cd exposure worldwide. At the 63rd JECFA meeting, using data from a meta-analysis conducted by EFSA [41], the obtained reference point of U-Cd was 5.24 µg/g Cr [42]. Then JECFA established a provisional tolerable monthly intake (PTMI) for Cd of 25 µg/kg body weight, which corresponds to a weekly intake of 5.8 µg/kg body weight. We believe it is necessary to continue this study to provide information that will contribute to future risk assessments.

Finally, we would like to emphasize the significance of the present results in the ongoing debate regarding the pathogenesis of Itai-Itai disease. This disease emerged in the early 20th century, and its recognition as a novel and puzzling illness among residents of the Jinzu River basin began with Dr. Noboru Hagino, who practiced medicine within this community for several generations [1,3]. As shown in Table 4, our study revealed that the incidence of renal tubular damage increased approximately twofold for every 1 g intake of Cd. This finding indicates that soil restoration efforts, which reduced Cd intake by 2 g, correspondingly decreased the incidence of renal tubular damage by a quarter. Together with the previous report by Sakurai et al. [7], our study strongly supports the conclusion that Cd is indeed the cause of Itai-Itai disease. Furthermore, this study is the first to demonstrate that health effects have decreased following the elimination of the causative environmental pollutant. This study presents a promising new research methodology that could be applied to various issues, including the pressing problem of air pollution.

One limitation of this study was that it was not feasible to conduct the survey targeting all residents from the beginning. It is evident that, due to the non-random nature of the sampling method employed, the results may not be entirely aligned with the prevailing exposure levels in the specified area. The possibility of selection bias cannot be discounted. Notwithstanding, the study design was initially adopted in accordance with the objective of the study, which was to elucidate the status of LCd intake and the health effects of Cd in these areas following soil restoration. In terms of Cd body burden, the LCd intake by Nogawa’s formula was developed to assess the amount of chronic intake from contaminated rice and other foodstuffs, subsequent to the identification of the effect of rice Cd concentration on the onset of Itai-itai disease and other adverse health outcomes. Nonetheless, the present study did not consider data pertaining to the consumption of river water. In this area, the installation of water supply systems occurred during the 1970s. Consequently, it is hypothesized that the impact of prior river water consumption was negligible in the outcomes of the present study. Other limitation of this study is that the long-time Cd exposure is assessed mainly by using the intake from rice based on the history of residence in the contaminated area as the LCd intake. There are other methods for estimating Cd intake, such as measuring Cd concentrations in food by duplicate method or detailed food surveys, and the method used in this study may have resulted in a rather rough estimate in terms of short-term exposure. The estimated rice intake (333.5 g/day) is based on data from the 1970s. However, data concerning fluctuations in rice consumption over the past several decades, categorized by gender, age and region is not available. Consequently, it is not possible to calculate more precise individual rice intake levels. On the other hand, for Cd exposure from the environment, once Cd enters the body, it is not easily eliminated, and it is very difficult to measure Cd exposure continuously over a long period using the above methods. Therefore, we believe that the method of this survey is the most appropriate to assess long-term exposure, taking into account changes in residential history, such as moving from one place to another. In addition, the relationship between LCd intake and U-Cd and U-β2MG concentrations was clearly demonstrated in this study, and we believe that it was primarily a reasonable indicator of Cd exposure.

## 5. Conclusions

In this study, we established that the relationship between LCd intake, U-Cd, and U-β2MG is a marker of renal tubular damage, allowing us to estimate exposure and indicators of renal effects in the absence of soil restoration. Our findings revealed that soil restoration significantly reduced Cd exposure, leading to decreased body burden and renal tubular effects. This shows, for the first time, the effectiveness of soil restoration as a countermeasure in Cd-polluted areas. We believe this information will be invaluable for developing future strategies to mitigate Cd exposure in polluted regions worldwide.

## Figures and Tables

**Table 1:** Characteristics of participants included in the regression model of urinary cadmium and β2-microglobulin.

	Men (*n* = 991)		Women (*n* = 828)	
Variable	Mean	SD ^1^	Mean	SD ^1^
Lifetime Cd intake, g	2.07	1.39	1.71	1.13
Age, years	60.4	15.9	58.8	17.0
Duration of residence in the polluted area, years	17.0	20.0	11.0	15.7
	GM ^2^	GSD ^3^	GM ^2^	GSD ^3^
U-Cd ^4^, µg/g Cr	0.98	2.15	1.53	2.24
U-β2MG ^5^, µg/g Cr	174	3.16	200	3.03
U-Cr ^6^, g/L	0.99	1.73	0.72	1.87
	N	%	N	%
U-β2MG ^5^ ≥ 300 µg/g Cr	203	20.5%	169	20.4%
U-β2MG ^5^ ≥ 1000 µg/g Cr	69	7.0%	55	6.6%
Nonsmoker	289	29.2%	707	85.4%
Ex-smoker	487	49.1%	96	11.6%
Smoker	215	21.7%	25	3.0%

^1^ Standard deviation. ^2^ Geometric mean. ^3^ Geometric standard deviation. ^4^ Urinary cadmium. ^5^ Urinary β2-microglobulin. ^6^ Urinary creatinine.

**Table 2:** Regression coefficients for an increase of 1 g in lifetime cadmium intake in relation to log-transformed urinary cadmium and β2-microglobulin.

U-Cd ^1^, µg/g Cr		Increase (95% CI ^2^)	*p*	R ^2^	VIF ^3^
Men (*n* = 991)	Lifetime Cd intake, +1 g	1.30-fold (1.27–1.34)	<0.001	0.287	1.015
	Ex-smoker (/nonsmoker ^4^)	1.40-fold (1.27–1.54)	<0.001		1.385
	Smoker (/nonsmoker ^4^)	1.20-fold (1.07–1.34)	0.002		1.374
Women (*n* = 828)	Lifetime Cd intake, +1 g	1.43-fold (1.37–1.49)	<0.001	0.244	1.000
U-β2MG ^5^, µg/g Cr		Increase (95% CI)	*p*		
Men (*n* = 991)	Lifetime Cd intake, +1 g	1.35-fold (1.29–1.42)	<0.001	0.133	1.000
Women (*n* = 828)	Lifetime Cd intake, +1 g	1.51-fold (1.42–1.60)	<0.001	0.172	1.000

^1^ Urinary cadmium. ^2^ 95% confidence interval. ^3^ Variance inflation factor. ^4^ Control category. ^5^ Urinary β2-microglobulin.

**Table 3:** Expected lifetime cadmium intake, urinary cadmium and β2-microglobulin assuming no soil restoration, and comparison with the actual levels.

	Men (*n* = 503)		Women (*n* = 342)	
	Mean (95% CI ^1^)	*p*	Mean (95% CI ^1^)	*p*
Expected lifetime Cd intake without soil restoration, g	5.06 (4.92–5.19)	<0.001	4.57 (4.41–4.73)	<0.001
Lifetime Cd intake, g	2.99 (2.88–3.11)		2.59 (2.47–2.72)	
Estimated decrease in lifetime Cd intake associated with soil restoration, g	2.06 (1.98–2.14)		1.98 (1.89–2.07)	
	GM ^2^ (95% CI ^1^)	*p*	GM (95% CI ^1^)	*p*
Expected U-Cd ^3^ without soil restoration, µg/g Cr	2.18 (2.09–2.26)	<0.001	4.22 (3.99–4.47)	<0.001
U-Cd ^3^, µg/g Cr	1.23 (1.16–1.31)		1.99 (1.85–2.14)	
U-Cd ^3^ divided by expected U-Cd ^3^ without soil restoration	0.57 (0.54–0.60)		0.47 (0.44–0.51)	
Expected U-β2MG ^4^ without soil restoration, µg/g Cr	430 (413–448)	<0.001	646 (605–689)	<0.001
U-β2MG ^4^, µg/g Cr	213 (191–238)		256 (223–294)	
U-β2MG ^4^ divided by expected U-β2MG ^4^ without soil restoration	0.50 (0.45–0.55)		0.40 (0.35–0.45)	

^1^ 95% confidence interval. ^2^ Geometric mean. ^3^ Urinary cadmium. ^4^ Urinary β2-microglobulin.

**Table 4:** Odds ratios and 95% confidence intervals of lifetime cadmium intake associated with β2-globulinuria.

U-β2MG ^1^ ≥ 300 µg/g Cr		Odds Ratio (95% CI ^2^)	*p*	Nagelkerke R ^2^
Men (*n* = 991)	Lifetime Cd intake, +1 g	1.75 (1.57–1.96)	<0.001	0.119
Women (*n* = 828)	Lifetime Cd intake, +1 g	1.84 (1.59–2.13)	<0.001	0.172
U-β2MG ^1^ ≥ 1000 µg/g Cr		Odds ratio (95% CI ^2^)	*p*	Nagelkerke R ^2^
Men (*n* = 991)	Lifetime Cd intake, +1 g	1.71 (1.47–2.00)	<0.001	0.158
Women (*n* = 828)	Lifetime Cd intake, +1 g	2.15 (1.76–2.64)	<0.001	0.129

^1^ Urinary β2-microglobulin. ^2^ 95% confidence interval.

## Data Availability

The data presented in this study are available on request from the corresponding author due to privacy restrictions.

## Abbreviations

The following abbreviations are used in this manuscript:

Cd	Cadmium
Cr	Creatinine
ICP-MS	Inductively coupled plasma mass spectrometry
LCd	Lifetime Cd
U-β2MG	Urinary β2-microglobulin
U-Cd	Urinary Cd
